# Microbiome-based therapeutics for metabolic disorders: harnessing microbial intrusions for treatment

**DOI:** 10.3389/fmedt.2025.1695329

**Published:** 2025-10-30

**Authors:** Nafees Ahmed, Vishwas Gaur, Madhu Kamle, Abhishek Chauhan, Ritu Chauhan, Pradeep Kumar, Namita Ashish Singh

**Affiliations:** 1 Department of Microbiology, Mohanlal Sukhadia University, Udaipur, Rajasthan, India; 2 Department of Botany, University of Lucknow, Lucknow, Uttar Pradesh, India; 3 Department of Biochemistry, University of Lucknow, Lucknow, Uttar Pradesh, India; 4 Amity Institute of Environmental Toxicology Safety and Management, Amity University, Noida, India; 5 Department of Biotechnology, Graphic Era (Deemed to be University), Dehradun, Uttarakhand, India; 6 College of Life Science & Biotechnology, Korea University, Seoul, Republic of Korea

**Keywords:** gut micobiota, dysbiosis, metabolic disorder, diabetes, FMT, vaginal microbiota

## Abstract

The rising global rates of metabolic disorders, such as obesity, type 2 diabetes, non-alcoholic fatty liver disease, and metabolic syndrome, call for new treatment methods beyond traditional drugs. The human gut microbiota, made up of trillions of microorganisms that plays a crucial role in maintaining metabolic balance through complex biochemical processes and interactions between hosts and microbes. Dysbiosis, which involves changes in microbial composition and a decrease in diversity, has become a major factor in metabolic problems. This disruption impacts the production of short-chain fatty acid, increase in permeability of intestine, and causes enduring low-grade inflammation. This review features into the potential of treatments based on microbiome for metabolic syndromes, focusing on probiotics, prebiotics, synbiotics, and postbiotics. It also encompasses innovative methods such as engineered microbial consortium, fecal microbiota transplantation (FMT), and vaginal microbiota transplantation (VMT). Probiotics show significant promise in improving blood sugar control and enhancing lipid levels. Prebiotics help bring about positive changes in microbial composition and the production of beneficial metabolites. Synbiotic combinations provide added benefits by helping good microbes thrive while supplying nutrients they can ferment. Postbiotics have recent research focus because they are safer, more stable, easier to store, and less likely to contribute to antibiotic resistance comparative to live probiotics. Even now there are substantial complications in translating microbiome research into standardized therapeutics despite of promising pre-clinical outcomes and some initial clinical data. These comprises individual variances, strain-specificity, dosage problems, regulation issues, and the necessity for personalised treatment strategies. Future success will depend upon personalized medicine, technological developments, and the incorporation of multi-omics strategy to generate metabolic health therapeutics depending on targeted microbiomes.

## 1 Introduction

The universal upsurge in the metabolic disorders which encompasses obesity, type 2 diabetes mellitus, non-alcoholic fatty liver disease, metabolic syndrome, and cardiovascular diseases, presents one of the major challenges in health care in the 21st century which substantially impacts the modern medication and public health policy. The number of adult persons suffering from diabetes globally has surpassed 800 million which is more than quadruple upsurge since 1990 (1). Furthermore, the prevalence of obesity among adults in the United States from August 2021 to August 2023 was 40.3% (2). This sharp intensification of the metabolic dysfunctions has sparked an urgent exploration for novel strategies of treatment beyond conventional drug therapies.

One of the greatest promising areas in this pursuit is the novel arena of treatments based on microbiome. This approach leverages the complex relationship between the gut microbiota and metabolic balance to create innovative treatment options. The human gut microbiota, made up of trillions of microorganisms in the gastrointestinal tract, is often referred to as a “forgotten organ.” When the microbiome is disturbed, it can greatly affect metabolism through various biochemical pathways and signalling networks (3). This microbial community contains about 100 times more genes than the human genome. It serves as a dynamic link between environmental factors, dietary components, and host physiology, managing vital metabolic processes. These include energy extraction, nutrient absorption, immune response, and hormone regulation.

The changes in gut microbiota composition and function, known as dysbiosis, are closely related to the development of metabolic disorders. This shift has changed our perspective and they are no longer seen only as issues within the host but as complex ecosystem imbalances involving interactions between hosts and microbes. The mechanisms behind these associations involve multiple interconnected pathways, such as altered short-chain fatty acid production, increased intestinal permeability causing metabolic endotoxemia, disrupted bile acid processing, changed incretin hormone release, and persistent low-grade inflammation (4).

The range of microbiome-based treatments includes probiotics (live helpful microorganisms), prebiotics (substances that support beneficial bacteria growth), synbiotics (mixtures of probiotics and prebiotics), and postbiotics (active compounds produced by probiotics). Advanced microbiome therapies that use engineered microbes have come forth as innovative solutions that can be programmed to generate specific therapeutic molecules on-site while responding to conditions within the gut environment (5).

Microbiome-based treatments have the potential to make a substantial economic difference by preventing disease progression, lowering reliance on medications, and promoting long-term metabolic health. This review summarizes the current understanding of microbiome-based therapies for metabolic disorders. It explores the vital connections among the metabolic health and gut microbiome which evaluates the therapeutic potential of several microbial intrusions, scrutinizes clinical trial outcomes, and looks forward to future advancements in this speedily changing arena. By evaluating both successes and challenges, this review enlighten researchers, healthcare providers, and policymakers about the immense potential of microbiome-based strategies while recognizing the considerable work needed to achieve their full therapeutic benefits in addressing the global rise in metabolic disorders.

## 2 Gut microbiota and its relation with metabolic disorders

The gastrointestinal tract of a human accommodates an intricate and dynamic ecosystem of microbes that are collectively labelled as the gut microbiota subsequently work as a crucial regulator of metabolism and health of the host. This complicated microbial community, encompassing more than 100 trillion microbial cells and rendering more than 1,000 distinct microbial species, sustains a sophisticated interdependent association with the human host (6). The gut microbiota has progressed to become a vital constituent of human physiology, prompting several metabolic processes involving immune function, metabolism of lipid, regulation of glucose, and energy homeostasis (7). Recent developments in microbiota study have discovered that modifications in composition and function of gut microbiota which is termed as dysbiosis, is intimately connected to various metabolic disorders pathogenesis, including type 2 diabetes, metabolic syndrome, obesity, and cardiovascular disease (7, 8).

Gut microbiota of the humans primarily comprises bacteria from four major phylum: Firmicutes, Bacteroidetes, Actinobacteria, and Proteobacteria. Firmicutes and Bacteroidetes constitutes 70%–90% of the overall microbial population (9). The phylum Firmicutes comprises microbial genera for instance *Lactobacillus*, *Clostridium*, *Ruminococcus*, and *Enterococcus* which generates short-chain fatty acids (SCFAs) (10). Bacteroidetes, primarily characterized by *Bacteroides* species, are specialized in breaking down complex carbohydrates and plant fibers (11).

The Firmicutes to Bacteroidetes (F/B) ratio has acquired recognition as a probable marker for metabolic health. Greater F/B ratios are usually found in individuals with obesity and metabolic problems (12). Dysbiosis associated with obesity is characterized by diminished microbial diversity, reduced abundance of beneficial taxa for instance *Bifidobacterium* species and *Akkermansia muciniphila*, and development of potentially harmful microbes (13). Type 2 diabetes is linked with decreased diversity of microbes and reduced abundance of butyrate producing microbes such as *Faecalibacterium prausnitzii*, *Eubacterium rectale*, and *Roseburia* species (7).

### 2.2 Factors influencing gut microbiota

The gut microbiome's function and composition are affected by an intricate mix of interior and exterior aspects which act over dissimilar timeframes. Comprehending these features is significant for generating targeted treatments for metabolic syndromes and enhancing therapeutic methods (14).

The most substantial changeable factor impacting the composition and metabolic results of the gut microbiota is diet. Fluctuations in diet can rapidly alter microbial communities within few hours to days (15). Western foods that are high in refined sugars, processed foods, and saturated fats, promotes the progression of potentially harmful microorganisms while restraining beneficial forms related to metabolic strength (16). Whereas, diets which consist of an assortment of dietary fiber, plant-based foods, and fermented products encourage microbial diversity and beneficial metabolites production (9). Dietary fiber serves as a chief food source for microbial fermentation, leads to synthesis of short-chain fatty acids (SCFAs) such as acetate, propionate, and butyrate (17). The mediterranean diet has unswervingly shown an association to superior microbial diversity and healthier metabolic consequences (7, 18).

Genetic factors of the host significantly impact the gut microbiota composition. Twin researches displays that around 10%–20% of microbial disparity comes from genetic factors (19, 20). The gut microbiome experiences momentous variations throughout an individual's life, with initial establishment at birth and variations through childhood, adulthood, and elderly phase (21). Environmental aspects such as geographical position, sanitation, climate, and exposure to contaminants impacts composition of gut microbiota (22). Choices of lifestyle, for instance stress levels, physical activity, sleep habits, and social networks also form microbial communities (23). Medicines particularly antibiotics have a robust influence on the gut microbiota 's composition and function, with effects that can sustain for months or even years (24).

### 2.3 Dysbiosis & metabolic disorders

Dysbiosis is an imbalance in the gut microbiome's diversity, composition, or metabolic action (25). This imbalance in microbiota is manifested by declined diversity, altered bacterial ratios, loss of advantageous bacteria, overgrowth of potentially damaging species, and reduced synthesis of helpful metabolites (26). The relationship among dysbiosis and metabolic dysfunction is a two-way connection; metabolic disorders might cause dysbiosis and on the other hand dysbiosis might worsen metabolic disorders (27).

Dysbiosis associated with obesity is characterized by diminished microbial diversity, augmented F/B ratio, reduced abundance of beneficial taxa for instance *Bifidobacterium* species and *Akkermansia muciniphila*, and development of potentially harmful microbes (13). *A. muciniphila* is a mucin degrading bacterium which constitutes 1%–5% of the gut microbiota in healthy persons, is consistently abridged in obesity and metabolic syndrome. Clinical researches have exhibited that abundance of *A. muciniphila* inversely associates with body mass index, inflammatory markers and resistance to insulin (28).

Type 2 diabetes is linked with distinctive variations in the composition and function of gut microbiome (29). Diabetic individuals show decreased diversity of microbes, changed representation of main bacterial taxa, and functional variations in metabolism of microbes (30). Specific changes encompass reduced abundance of butyrate producing microbes such as *Faecalibacterium prausnitzii*, *Eubacterium rectale*, and *Roseburia* species accompanied by augmented depiction of opportunistic pathogens and pro-inflammatory microbes (7).

Non-alcoholic fatty liver disease (NAFLD) is connected to dysbiosis of gut microbiota via the gut-liver axis (31). Alterations by dysbiosis accord to pathogenesis of NAFLD through several mechanisms involving upsurged permeability of intestines causing the portal circulation of microbial LPS and other inflammatory intermediaries, changed metabolism of bile acid, and dysregulated metabolite production (7). Novel pathways for therapeutic intercessions have been unlocked through understanding of the mechanisms of metabolic dysfunction allied with dysbiosis (27).

## 3 Role of probiotics, prebiotics, synbiotics and postbiotics in regulating gut microbiota and prevention of metabolic disorders

### 3.1 Probiotics, prebiotics, synbiotics and postbiotics

Kollath defined probiotics as active substances that perform critical functions for health (32). In 2001, the Food and Agriculture Organisation (FAO) and the World Health Organisation (WHO) given a definition, characterising probiotics as “live microorganisms which, when administered in adequate amounts, confer a health benefit to the host” (33, 34). Probiotic bacteria are often classified into two types: conventional and non-conventional. *Lactobacillus*, *Streptococcus*, *Escherichia*, and *Bifidobacterium* are examples of conventional strains, whereas non-conventional strains like *Akkermansia*, *Faecalibacterium*, *Eubacterium*, *Roseburia*, *Christensenella*, and *Clostridium* have recently received attention for their health-promoting potential (35).

Probiotics have a variety of beneficial effects, including competitive exclusion of pathogens, production of antimicrobial compounds, improvement in intestinal barrier functions, immunomodulation, and modulation of the gut-brain axis (36, 37, 38). They compete for nutrients and adhesion sites, secrete antimicrobial compounds such as SCFAs, organic acids, hydrogen peroxide (39), and bacteriocins (40), and strengthen gut integrity by upregulating mucin and tight junction proteins such as occludin and claudin-1 (41, 42). Probiotics modulate innate and adaptive immunity by modifying dendritic cells, macrophages, B and T lymphocytes, and boosting anti-inflammatory cytokines. While probiotics have long been acknowledged for their role in gut health, more emphasis is now being paid to prebiotics, which are dietary components that nourish and promote the activity of these beneficial microbes (43).

Gibson and Roberfroid created the notion of prebiotics in 1995 (44). Prebiotics are defined as “substrates which are selectively utilised by host microbes conferring a health benefit” (45). Prebiotics are non-digestible dietary components which helps the host by selectively augmenting the growth and/or activity of certain intestinal flora. Inulin, fructooligosaccharides (FOS), galactooligosaccharides (GOS), and lactulose are examples of common prebiotics (46, 47). Prebiotic fermentation synthesizes short-chain fatty acids (SCFAs) such as acetate, butyrate, and propionate. These SCFAs lower gut pH from ∼6.5 to ∼5.5, inhibiting pathogenic bacteria and promoting beneficial microbes (48, 49).

Individual gut microbiota differences, dosing challenges, gastrointestinal side effects, short shelf life, and safety concerns for immunocompromised individuals, particularly under processing conditions such as pasteurisation or baking, limit probiotic effectiveness (50, 51).

To address the limits of standalone probiotics and prebiotics, synbiotics have emerged as a viable technique that mixes the two to provide greater health advantages. In 1955, Gibson and Roberfroid (44) proposed synbiotics, which are cooperative concoctions of probiotics and prebiotics envisioned to enhance survival, colonisation, and activity of the probiotic in the gut (52, 53). Synbiotics enhance implantation and function by specifically stimulating helpful bacteria, overcoming difficulties like as pH and oxidative stress, both of which restrict probiotic viability (54).

Postbiotics have arisen as a current research focus because they are safer, more stable, easier to store, and less likely to contribute to antibiotic resistance than live probiotics. As stated by the International Scientific Association for Probiotics and Prebiotics (ISAPP), postbiotics are “preparations of inanimate microorganisms and/or their components that confers a health benefit on the host” (50). Postbiotics improve health by inhibiting microorganisms, improving the intestinal barrier, and modifying immune responses via interactions with host receptors such as TLRs and NLRs (55).

While probiotics and prebiotics each provide significant health benefits, synbiotics combine their strengths to produce synergistic effects, and postbiotics provide a safer, more stable alternative with bioactive components that influence host health without the risks associated with live microorganisms (55).

### 3.2 Impact of diet & probiotics, prebiotics, synbiotics and postbiotics on Gut Microbiota

The gut microbiota, with roughly 3.8 × 10¹³ microorganisms, outnumbers human cells and plays a crucial role in supporting host health (56, 57). This intricate micro ecology which includes bacteria, yeasts, viruses, and parasites categorised into five primary phyla: Firmicutes, Bacteroidetes, Actinobacteria, Proteobacteria, and Verrucomicrobia. Firmicutes and Bacteroidetes account for over 90% of the total microbial population (57, 58). Gut microbiota composition varies across individuals and is determined by factors such as age, genetics, birth mode, infant feeding practices, antibiotic usage, geography, and, most importantly, food (59). Diet has a bi-directional relationship with the microbiota, influencing nutrient absorption and metabolism (60).

Diet has a strong influence on the gut microbiome through macronutrients, micronutrients, and bioactive chemicals, influencing microbial composition, diversity, and function (61). Dietary fibre influences the gut microbiota, especially by boosting the abundance of SCFA-producing bacteria (62). Fibres like inulin, guar gum, resistant starch, galacto-oligosaccharides, fructo-oligosaccharides, and arabinoxylan oligosaccharides consistently promote beneficial microbes like *Bifidobacterium*, *Faecalibacterium*, *Ruminococcus*, *Lactobacillus*, *Akkermansia*, and *Roseburia* (63). Recent research indicates that high-protein diets increase gut microbial diversity and modify microbiota composition differently from normal-protein diets, enriching *Akkermansia* and *Bifidobacterium* while decreasing *Prevotella* (62, 58). High-fat diets, particularly those high in saturated fats, promote dysbiosis by boosting Firmicutes and Proteobacteria while lowering Bacteroidetes (63, 64, 57).

Probiotics regulate the synthesis of gastrointestinal hormones such as leptin, ghrelin, and GLP-1, which helps with hunger regulation and metabolic health (65). They create short-chain fatty acids from dietary fibre fermentation and produce organic acids and bacteriocins that inhibit infections (66). *Bifidobacteria* form acetate, which helps other gut microbes thrive. *Bifidobacteria* is one of the most profuse and substantially functional group of microbes in healthy individual's microbiome, primarily in newborns where they encompass approximately 90% of total microbiota (67). Prebiotics have a good effect on gut health by suppressing type 2 T helper immune responses and boosting calcium absorption by creating SCFAs through fermentation (46, 47). Synbiotics can assist regulate the gut microbiota during weight loss and improve blood glucose, lipid profiles, and body weight in T2DM patients (68, 69). Postbiotics boost gut and metabolic health by regulating immunity and improving glucose and insulin metabolism while avoiding the hazards associated with live bacteria (50).

### 3.3 Microbiome based metabolic treatment

**Figure 1:**
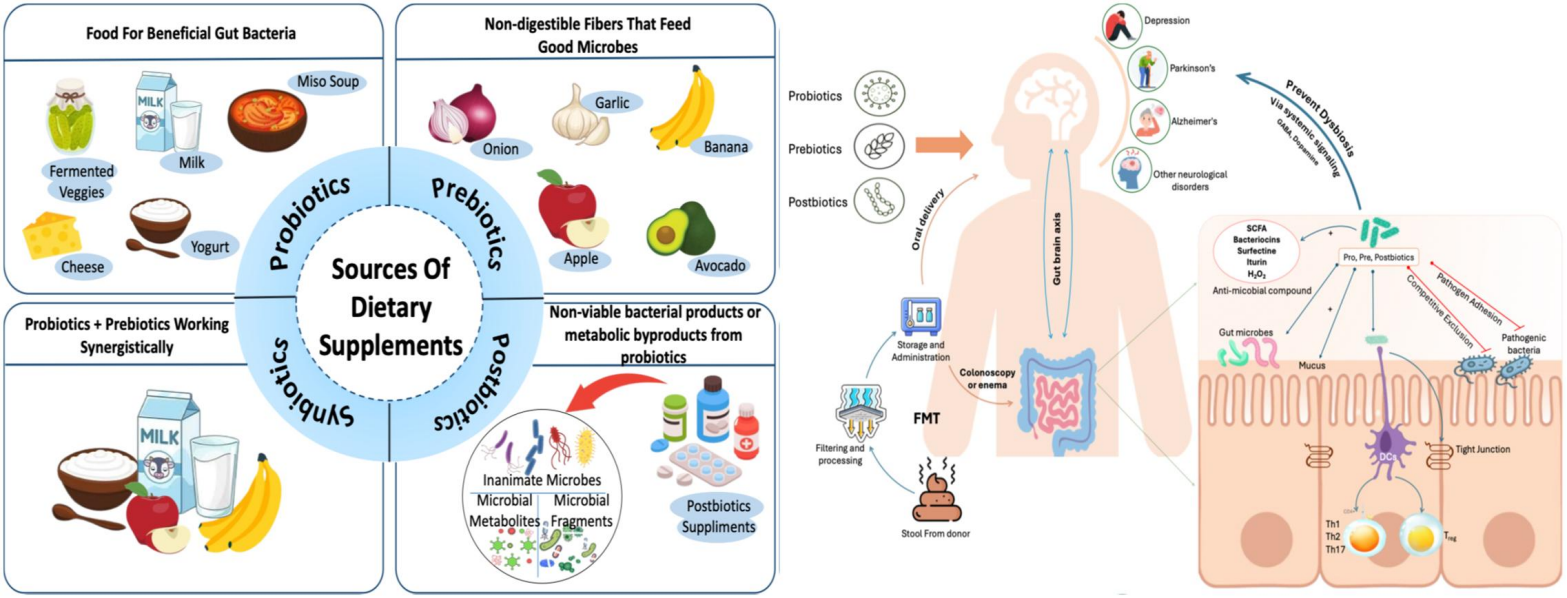
Sources of dietary supplements and their mechanism in regulating gut-brain axis.

The gut microbiome is defined as the collective genomes of all bacteria living in the gut. A change in gut microbiome homeostasis caused by changes in genetics, nutrition status, lifestyle, and other factors can lead to microbiome dysbiosis, which in turn results in chronic diseases including inflammatory bowel disease (IBD) (70), cardiovascular disease (71), neurological diseases such as autism and Parkinson's, metabolic conditions such as obesity and diabetes, and certain cancers type 2 diabetes (T2D), metabolic dysfunction-associated steatotic liver disease (MASLD), hypertension, and hyperlipidemia, all of which are increasingly prevalent worldwide (72). Several microbiome-based treatment techniques have emerged as viable approaches for treating metabolic and inflammatory illnesses. These include probiotics, prebiotics, synbiotics, and faecal microbiota transplantation (FMT) as depicted in Figure 1.

#### 3.3.1 Probiotics, prebiotics, and synbiotics treatment technique

Prebiotics, probiotics, synbiotics (combinations of the two), and postbiotics are important microbiome-based treatments for treating metabolic diseases as shown in Table 1. While they may have potential benefits, such as enriching beneficial microorganisms, enhancing gut barrier function, and altering host metabolism, clinical evidence of their safety and efficacy is sparse. Probiotics frequently encounter colonisation resistance, exhibit strain and site-specific effects (73). Additionally, a study using an *in vitro* gut model with fecal samples from obese donors showed that synbiotic supplementation with *Limosilactobacillus reuteri* KUB-AC5 and *Wolffia globosa* powder increased beneficial bacteria, decreased *Enterobacteriaceae*, and enhanced levels of butyrate while lowering detrimental p-cresol (74). Postbiotics and synthetic microbes are developing alternatives with promising metabolic benefits, but they face challenges such as complex host-microbe interactions, unclear metabolic pathways, and tailored delivery.

**Table 1:** Probiotics, prebiotics, synbiotics, and postbiotics in treating metabolic illness.

Supplements	Target disease	Doses	Mechanisms and effects	References
*Bifidobacterium lactis LMG P-28149* and *Lactobacillus rhamnosus LMG S-28148*	Obesity and insulin resistance	10^8^ CFU	*Rikenellaceae* and *Akkermansia* muciniphila restoration. Lipoprotein lipase and PPAR*γ* expression are upregulated. TG clearance and insulin sensitivity enhancement A reduction in *Lactobacillaceae*	(75)
*Lactobacillus acidophilus, L. rhamnosus, Bifidobacterium bifidum, B. longum, Enterococcus faecium*	Depression and Anxiety in pregnant women with Gestational Diabetes Mellitus (GDM)	—	Gut-Brain Axis modulation, Anti-inflammatory effect, Enhanced adherence to the Mediterranean diet	(76)
* Lactiplantibacillus plantarum* YC17	Fatty liver disease	—	Enhances FFAs esterification, increases IPA and IAA, activates Ahr pathway, promotes P53 degradation, Increases beneficial gut bacteria (*Lactobacillus*, *Clostridium*)	(77)
* Phocaeicola dorei*	Metabolic dysfunction-associated steatotic liver disease (MASLD)	10⁹ CFU	Enhances *β*-oxidation gene expression, modulates bile acids, reduces TNF-α and CXCL10, inhibits inflammation and cell proliferation, Reduces liver lipid accumulation, alleviates MASLD progression, lowers inflammation, and improves liver histology	(78)
* Lactobacillus pantheris TCP102*	Cancer	—	Immune-enhancing activity and inhibition of cancer cell proliferation, EPSs stimulated NO, TNF-α, and IL-6 production in macrophages	(79)
*Lactobacillus and Bifidobacterium species*	Ulcerative colitis	3 × 10^10^	Increases IL-10, reduces C-reactive protein and IgA, modulates immune response and gut inflammation	(80)
* Lactobacillus acidophilus, Bifidobacterium bifidum, Bifidobacterium longum and selenium*	Alzheimer's disease	—	Regulation of metabolic abnormality and oxidative stress, Reduced serum hs-CRP, Reduced serum triglyceride, Increased GSH, Increased antioxidant.	(81)
*Lactobacillus plantarum Lp62*	Bacterial vaginosis	—	Reduced *G. vaginalis* load, no leucocyte recruitment, lowered vaginal cytokines, normalized cytokine gene expression	(82)

#### 3.3.2 Faecal microbiota transplantation technique

FMT includes introducing stool from a healthy donor into a patient's gastrointestinal tract to restore gut bacteria equilibrium. It is now a well-established treatment for recurrent Clostridioides difficile infection (CDI), with cure rates of up to 90%. Beyond CDI, FMT is being studied for its therapeutic potential in inflammatory bowel disease (IBD), with clinical studies indicating that it can reduce gut inflammation and microbial composition (83, 84). Le et al. found that FMT resulted in durable increases in gut microbial diversity and decreased pathogenic taxa in paediatric ulcerative colitis (85). Similarly, Shekar et al. found that FMT may assist Parkinson's patients by increasing the production of beneficial metabolites like short-chain fatty acids (SCFAs), promoting gut-brain axis activity, and potentially decreasing disease progression (86).

An important variable in mental health, the microbial-gut-brain (MGB) axis, is modulated by FMT, providing a novel treatment for depression. FMT restores a healthy microbial ecosystem by addressing dysbiosis in the gut microbiota milieu. This affects important targets such the NLRP3 inflammasome and Sig-1R, which are linked to neuroinflammatory and neurochemical pathways linked to depressive disorders. Furthermore, FMT can enhance the antidepressant potential by utilizing the medicinal qualities of advantageous herbs (87).

#### 3.3.3 Vaginal microbiota transplantation

The vaginal microbiota has a somewhat lesser diversity of microorganisms compared to the intestinal tract. A wide variety of lactic acid bacteria largely control the vaginal environment in order to preserve homeostasis (88). In addition to fluctuating throughout pregnancy and menopause, the vaginal microbiota's composition can also alter dynamically over shorter timescales of days to months (89, 90).

The vaginal microbiota of pre-term birth (PTB) showed that the PTB group had a significantly higher proportion of harmful bacteria (such as Desulfovibrionaceae, Helicobacter, and Gardnerella) and a significantly lower proportion of beneficial bacteria (such as Lactobacillus, Ruminococcus, and Megamonas). This difference was closely linked to the blood's biochemical parameters (white blood cells, neutrophils, NLR, and SIRI (91).

Vaginal Microbiota Transplantation (VMT) aids in sustaining the vaginal acidity and hinder the pathogenic bacteria by restoring the central Lactobacillus species. Inhibiting the NF-*κ*B signaling pathway is one of its main strategies, which lowers inflammatory cytokines like TNF-α, IL-1β, and IL-6. This promotes healing and lessens tissue inflammation. In addition to being more biocompatible than antibiotics, VMT can work better when combined with other therapies. VMT is a promising noninvasive approach to the treatment of endometritis, with safety and microbial benefits. With its safety and microbiological advantages, VMT is a viable noninvasive treatment for endometritis (92).

The safety and effectiveness of VMT in treating bacterial vaginosis, recurring yeast infections, and other vaginal disorders have been shown in numerous studies. Additionally, the technique has demonstrated encouraging outcomes in lowering pregnant women's risk of premature birth and sexually transmitted diseases. For women who have ongoing vaginal issues, VMT is a minimally invasive, safe, and effective therapy alternative (93).

Compared to babies born vaginally, babies born via cesarean section (C-section) frequently have a different gut flora and are more susceptible to atopic and immune-related disorders (94). *Bifidobacterium*, *Bacteroides*, and *Parabacteroides* are generally more abundant in vaginally born newborns than in C-section babies (95). Human milk oligosaccharide (HMO) breakdown is frequently accelerated by these first colonizers, leading to the generation of short-chain fatty acids (SCFA) and colonization resistance which shape the microbiome and immune system leading to a healthier life (96, 97). On the other hand, skin and hospital-associated bacteria such *Staphylococcus*, *Enterococcus*, *Klebsiella*, and *Clostridium* species (95, 98) frequently invade newborns born after cesarean section. These are more likely to have genes for antibiotic resistance and frequently lack the capacity to break down HMOs or generate SCFAs (99).

#### 3.3.4 Artificial microbial consortia technique

Emerging personalized strategies such as engineered microbial consortia are gaining attention for targeted microbiome-based interventions in metabolic disorders. Artificial microbial consortia (AMCs) are precisely designed communities of microorganisms tailored to modulate the gut microbiota and address specific pathological states. These consortia could include naturally helpful microorganisms or genetically engineered strains with higher medicinal potential. AMCs involve the deliberate selection and assembly of microbial strains with specific metabolic, immunomodulatory, and ecological roles (100). A study found that co-administration of *Bifidobacterium pseudocatenulatum* JJ3 and the engineered strain BsS-RS06551 significantly reduced obesity and associated metabolic dysfunctions in high-fat diet-induced obese mice (101).

#### 3.3.5 Precision dietary modulation

Precision dietary modulation uses personalised nutritional therapies to target the gut microbiota and takes into account individual-specific characteristics such as genetics, dietary patterns, lifestyle behaviours, and metabolomic signatures (102). This approach tailors dietary advice to prevent and manage metabolic and gastrointestinal illnesses (103). Despite its potential, the application of precision nutrition is hampered by hurdles such as the complexities of microbiome analysis and an imperfect understanding of causative microbiome-health interactions.

#### 3.3.6 Clinical trials: success and failure

Clinical trials are critical in turning preclinical microbiome research into therapeutic applications, providing a controlled environment to assess safety, effectiveness, and mechanistic outcomes in human populations. However, the effects of microbiome-based therapies in metabolic illnesses have varied, indicating the complex and individualised nature of host-microbe interactions.

Faecal microbiota transplantation (FMT) has showed promise for improving metabolic parameters. Wu et al. found that both FMT alone and FMT combined with metformin significantly improved insulin resistance (HOMA-IR), body mass index (BMI), and glycaemic management in a randomised clinical trial comprising 31 newly diagnosed type 2 diabetic mellitus (T2DM) (104). These approaches resulted in successful donor microbiota colonisation, enhanced microbial diversity, and modification of more than 200 microbial species. Notably, *Bifidobacterium adolescentis* and *Synechococcus* sp. were adversely linked with HOMA-IR, which highlights the therapeutic potential of microbiota manipulation in T2DM therapy. Similarly, targeted probiotic and synbiotic formulations have shown moderate but significant improvements in metabolic health. Othman et al. conducted a clinical trial on obese people, comparing food alone to prebiotic (carob) or probiotic treatment in 45 participants. While all groups lost weight, the prebiotic and probiotic groups saw greater increases in fat mass loss, muscle strength, insulin sensitivity, sleep quality, and psychological well-being (105). In a randomized controlled trial, the FMT from healthy individual into a patient suffering from IBS with mild to modest depression and anxiety, exhibited alleviation in anxiety and depression after treatment along with IBS symptoms, leading to substantial improvement in the quality of life (106). Adult patients suffering from Major Depressive Disorder (MDD) on undergoing FMT from healthy donor showed significant augmentation in mean gastrointestinal symptom scores and demonstrated greater enhancements in quality of life measures (107).

Despite these positive findings, many restrictions remain. A randomised trial by (NCT03125564) evaluating FMT in diarrhoea-predominant IBS patients found no significant improvement in overall IBS severity, but showed notable improvement in bloating symptoms (72% vs. 30%, *p* = 0.005), linked to reduced hydrogen sulphide-producing bacteria and microbial changes (e.g., ↓*Ruminococcus gnavus*, ↑*Lawsonibacter*) (108). Many clinical trials are unclear about probiotic strain specification, dosing regimen, and product formulation, with over 1,000 trials failing to publish standardised product information (109, 110).

## 4 Limitations, challenges and future outlook

The arena of microbiome-based therapeutics for metabolic disorders has observed remarkable advancement over the past decade, transitioning from conceptual bases to clinical realities with recent FDA approvals of live biotherapeutic products like Rebyota™ and Vowst™ (111). However, despite these substantial milestones, the translation of microbiome research into therapeutic interventions for metabolic ailments faces several complex challenges spanning scientific, technological, regulatory, and clinical domains.

One of the biggest challenges in microbiome-based treatments is the large differences in gut microbiome composition and function among individuals (112). The human gut microbiota shows significant diversity, which is affected by genetics, diet, lifestyle, environmental factors, and medical history (6, 113). This variation makes it hard to create standardized therapies that work well for all patients. Although many studies show links between changes in the microbiome and metabolic disorders, proving direct cause-and-effect relationships is tough (5). The lack of standard methods in microbiome research creates major hurdles for applying findings clinically, as differences in sample collection, processing, sequencing methods, and analysis can influence results and hinder reproducibility (111).

The rules around microbiome-based therapies are complex and change often, creating serious issues for developing and selling products. Engineering microbiome-based therapies is tricky, especially when it comes to keeping live products viable, stable, and consistent. Quality control must ensure the identity, purity, strength, and safety of microbes, while also maintaining consistency between batches (111). Safety assessments need to thoroughly evaluate both direct and indirect risks to the host, which includes concerns about microbial transfer, development of antibiotic resistance, and reactions from the immune system (114).

Forming clinical trials for treatments based on microbiome presents exclusive operational complications (5). Patient stratification strategies are important for identifying those who are most expected to benefit from definite treatments, but discovering dependable biomarkers is still a difficulty (115). Educating healthcare providers and gaining patient acceptance are additional hurdles for implementation.

The future of microbiome-based treatments will be influenced by several new technological advancements. Artificial intelligence and machine learning can help spot complex patterns and create predictive models for treatment response and customized therapy selection (115). Precision medicine based on individual microbiome profiles, genetic factors, and metabolic status will likely take center stage in future development. Multi-omics approaches that combine microbiome, metabolomic, proteomic, and genomic data may provide better targeting for therapies (116).

The future success of microbiome-based therapies for metabolic disorders will rely on ongoing scientific innovation, changes in regulation, and cooperation across academia, industry, and healthcare (115).
