# Correction: Microbiome-based therapeutics for metabolic disorders: harnessing microbial intrusions for treatment

**DOI:** 10.3389/fmedt.2025.1736962

**Published:** 2025-11-28

**Authors:** Nafees Ahmed, Vishwas Gaur, Madhu Kamle, Abhishek Chauhan, Ritu Chauhan, Pradeep Kumar, Namita Ashish Singh

**Affiliations:** 1 Department of Microbiology, Mohanlal Sukhadia University, Udaipur, India; 2 Department of Botany, University of Lucknow, Lucknow, India; 3 Department of Biochemistry, University of Lucknow, Lucknow, India; 4 Amity Institute of Environmental Toxicology Safety and Management, Amity University, Noida, India; 5 Department of Biotechnology, Graphic Era (Deemed to be University), Dehradun, India; 6 College of Life Science & Biotechnology, Korea University, Seoul, Republic of Korea

**Keywords:** gut microbiota, dysbiosis, metabolic disorder, diabetes, FMT, vaginal microbiota

Author affiliation

Authors affiliations were erroneously assigned. The following affiliations are added:

Amity Institute of Environmental Toxicology Safety and Management, Amity University, Noida, India for author Abhishek Chauhan.

Department of Biotechnology, Graphic Era (Deemed to be University), Dehradun, Uttarakhand, India for author Ritun Chauhan.

The following affiliations are being removed:

for author Abhishek Chauhan Department of Biotechnology, Graphic Era (Deemed to be University), Dehradun, Uttarakhand, India.

for author Ritu Chauhan Amity Institute of Environmental Toxicology Safety and Management, Amity University, Noida, India.

The original version of this article has been updated.

